# The wheat stripe rust resistance gene *YrNAM* is *Yr10*

**DOI:** 10.1038/s41467-024-47513-z

**Published:** 2024-04-17

**Authors:** Katherine Dibley, Matthias Jost, Robert McIntosh, Evans Lagudah, Peng Zhang

**Affiliations:** 1 CSIRO Agriculture and Food, Commonwealth Scientific and Industrial Research Organisation, GPO Box 1700, Canberra, ACT 2601 NSW Australia; 2 The University of Sydney, School of Life and Environmental Sciences, Plant Breeding Institute, Cobbitty, NSW 2570 NSW Australia

**Keywords:** Plant immunity, Biotic, Plant genetics, Agricultural genetics

**arising from** F. Ni et al. *Nature Communications* 10.1038/s41467-023-39993-2 (2023)

The wheat stripe rust resistance gene *Yr10* is defined by a unique array of responses to multiple isolates of the stripe rust pathogen *Puccinia striiformis* f. sp. *tritici* (*Pst*), yet has been attributed to two different genes in the wheat host. Sequencing of putative *Yr10* mutants by Ni et al. identified changes in a gene named *YrNAM* that was closely linked to gene *Yr10*_*CG*_, reported earlier as the *Yr10* candidate. Independent analysis of mutants in a universal reference *Yr10* stock by us identified changes in *YrNAM*. An agreed simplification of two separate gene identities conferring a common specificity to a single *Yr10* designation will benefit the wider cereal research community.

*Yr10* was originally identified in Turkish wheat landrace PI 178383 and is located on chromosome arm 1BS^1^. It was first deployed in the cultivar (cv.) Moro (CI 013740) in the Pacific Northwest, USA^2^. Moro and a Moro-derived near isogenic line (NIL) Avocet S + Yr10 (Avocet S*6/Moro)^3^ were globally distributed and continue to be included in differential sets for phenotyping *Pst* in Australasia, Europe, India and North America. Two publications reported the putative cloning of *Yr10* as homologs of two closely linked genes, *TraesCS1B03G0000200*, an NLR designated *Yr10*_*CG*_, Genbank accession AF149112^4^, and more recently homologs of *TraesCS1B03G0003600 LC.1* and *TraesCS1B03G0003500 LC.1*, Genbank accession OP490604, was designated *YrNAM*^5^. We believe this situation is untenable and that one of these linked genes cannot be *Yr10*. Here, we present evidence that *YrNAM* is *Yr10*.

The CC–NBS–LRR candidate gene *Yr10*_*CG*_ was first identified following RAPD analysis and genetic mapping, and was used in genetic analysis, gene silencing and transgenesis to demonstrate putative functionality^4^. The identified sequence was present in Moro and several backcross derivatives predicted to have *Yr10*, along with cv. Jacmar and *Triticum spelta* 415 shown earlier to carry *Yr10*^6^. Of 46 plants recovered from scutella bombarded with the *Yr10*_*CG*_ partial genomic clone 4B, only one transgenic individual produced a Moro-like stripe rust response with a *Yr10-*avirulent *Pst* isolate, and the same plant was susceptible to a *Yr10-*virulent isolate. Reliance on one out of 46 independent transgenic events as a basis for identifying *Yr10*_*CG*_ as the causal gene for *Yr10* is however inadequate and does not allow for any effects of somaclonal variation. Subsequent tests from an independent study, carried out on additional *Yr10*_*CG*_ cDNA transgenic events, failed to detect the *Yr10* resistance phenotype^7^. Virus induced gene silencing of *Yr10*_*CG*_, while suggesting the possible requirement of *Yr10*_*CG*_ or similar sequence in stripe rust resistance in the specific host used, does not address the sufficiency of *Yr10*_*CG*_ as the causal gene of *Yr10*-mediated resistance.

In a subsequent study^7^, the *Yr10*_*CG*_ sequence AF149112 was mapped and shown to be located 1.2 cM from *Yr10*, hence indicating that it was not *Yr10*. Of 62 wheat lines identified with an AF149112-specific marker, only 10 gave stripe rust responses typical of Moro. In a further experiment, all T1 progeny of cv. Bobwhite transformed with the AF149112 sequence were susceptible to a *Pst* isolate avirulent on Moro. This publication provided strong evidence that the AF149112 sequence was not the causal gene for *Yr10*.

More recently, Ni et al.^5^ generated 12 putative *YrNAM* mutants in two homozygous lines identified in a cross of Moro and a susceptible line. Seven of these mutants were subjected to RNA-seq and transcriptome analysis, and mutations were identified in a gene designated *YrNAM*, which was located 1.2 cM from the *Yr10*_*CG*_ sequence. *YrNAM* encodes a non-canonical resistance protein with a NAM (No Apical Meristem) domain and a ZnF (Zinc Finger)-BED domain at the N and C termini, respectively^5^. It is structurally similar to *Rph7*, a leaf rust resistance gene in barley containing NAC transcription factor (TF) and ZnF-BED domains^8^. Together YrNAM and Rph7 represent a different class of R proteins, which we propose be designated as Triticeae NAC-BED domain (TNB) proteins. Although only two resistance genes have been reported with this structure to date, NAC TF domains feature widely as regulators of plant immunity^9^, and several other cloned resistance genes possess ZnF-BED domains (e.g. *Rph15* in barley^10^ and *Yr5a*, *Yr5b*, and *Yr7* in wheat^11^) albeit in a different clade to that of TNB proteins. It thus appears likely that additional TNB resistance genes remain to be discovered.

All 12 YrNAM mutants were confirmed to encode non-synonymous amino acid changes in the same gene; six in each of the NAM and ZnF-BED protein domains^5^. Concurrent with that study, we produced four EMS-derived *Yr10* mutants in Avocet S + Yr10 (Fig. 1). All four mutations were in *YrNAM* (Fig. 1b, c) and not in *Yr10*_*CG*_, with supporting evidence from mutational genomics of enriched NLR sequences (MutRenSeq) including AF149112 previously reported to encode *Yr10*_*CG*_. MutRenSeq analysis did not detect any mutations in *Yr10*_*CG*_. This corroborated the previous high resolution mapping study distinguishing *Yr10* from *Yr10*_*CG*_^7^, and subsequent validation by *YrNAM* transgenesis^5^. Interestingly, three of the susceptible mutants coincided with those reported by Ni et al.^5^ at amino acid positions G193E (independent mutants M6225 & M6227) and D155N (M6211) whereas a fourth mutation (M6220) is a putative intron splice junction mutation presumably affecting the last exon with the ZnF-BED domain. Amino acid conservation values were calculated using the EVcouplings server (https://evcouplings.org)^12^ on 6268 NAM domain-containing sequences retrieved and aligned with Yr10. The G193E mutation occurs at a very highly conserved amino acid, G193 (Fig. 1d), with 97.2% conservation across the set of 6282 homologs, and this together with the independent generation of loss-of-function mutations in the study by Ni et al.^5^ for this residue indicate that G193 plays a critical role in NAM domain function.

**Fig. 1:**
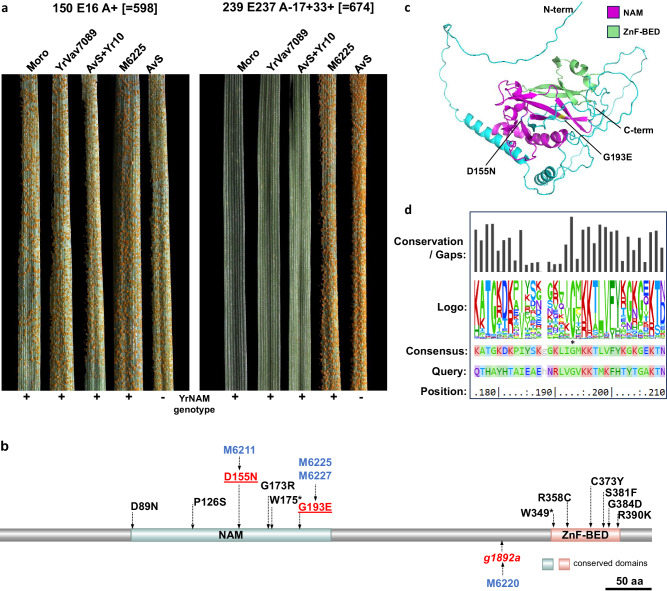
YrNAM mutations support identity as Yr10. **a** Seedling leaves of cv. Moro, YrVav7089 (backcross derivative of *Triticum vavilovii* AUS22498), AvS+Yr10, M6225 (AvS+Yr10 mutant), and Avocet S (AvS) infected with *Yr10* virulent*-* (culture no. 598, race 150 E16 A +) (left) and *Yr10-*avirulent (culture no. 674, race 239 E237 A-17 + 33 +) (right) *Puccinia striiformis* f. sp. *tritici* isolates. All five lines were susceptible to the *Yr10-*virulent isolate. Moro, YrVav7089, and AvS+Yr10 were resistant whilst mutant M6225 and AvS were susceptible to the *Yr10-*avirulent isolate, respectively. **b** Schematic of the protein structure of *Yr10* (redrawn from Ni et al.^5^), which includes NAM and ZnF-BED protein domains. Our EMS-induced AvS+Yr10 mutation line numbers are in blue and non-synonymous amino acid substitutions are in red, while the intron splice junction mutation base substitution is given in italicized red. Underlined mutations were also independently generated and reported by Ni et al.^5^ Mutations in black are non-synonymous mutations reported for *YrNAM*^5^. The mutation sites given are relative to ‘ATG’ for nucleotides, or methionine for amino acids. **c** AlphaFold-predicted structure of Yr10 and positions of mutations D155N (blue residue) and G193E (yellow residue). NAM and ZnF-BED domains are colored magenta and green, respectively. **d** EVcouplings output across a window centered on highly conserved amino acid G193 (marked by asterisk) showing sequence conservation, sequence logo and consensus sequence for a set of 6268 putative NAM-domain containing proteins. G193 is conserved across 6090 of the 6268 sequences retrieved. Source data are provided as a Source Data file.

There were apparent problems concerning the phenotyping of transgenic plant stripe rust responses, as wildtype Fielder was not fully susceptible as expected for an isolate of *Pst* race CYR34^5^. We suggest that was due to technical problems associated with the experiments rather than suggested combined effects of defeated genes *Yr6* in Fielder and *Yr9* in CB037 as proposed by Ni et al.^5^.

Ni et al.^5^ observed that *YrNAM* was rare in the wheat gene pool. Our results, based on rust response phenotyping with the recent and differentiating *Yr10*-avirulent (culture no. 674, race 239 E237 A-17 + 33 +) and *Yr10*-virulent (culture no. 598, race 150 E16 A +) *Pst* isolates and *YrNAM* gene-specific sequence assays, confirmed that *Yr10* is present in Iranian accession *T. spelta* 415 (Plant Breeding Institute accession C89.19)^6^, and *T. vavilovii* AUS22498^13^ and four backcross derivatives (Fig. 1a). Ni et al.^5^ also suggested that the widespread absence of *YrNAM* in contemporary wheat cultivars was due to its close linkage with the brown glume color, considered as an undesirable trait. There is no evidence that gene *Rg1* for red/brown glumes causes negative yield or grain quality effects, rather its absence appears attributable to cosmetic factors of breeder and/or farmer choice. Due to the close linkage of *Rg1* and *Yr10* in accession PI 178383, most cultivar derivatives of this accession will have brown glume color; however, *Rg1* is not present in *T. spelta* 415 and *T. vaviloii* AUS22498.

In conclusion, the current evidence indicates that *Yr10* is *YrNAM*, a gene with Triticeae NAC-BED domain (TNB) domain architecture similar to that reported for barley leaf rust resistance gene *Rph7*^8^ Together, these genes represent a different class of R proteins. We propose the simplification of two separate gene identities conferring a common specificity to a single designation, *Yr10*, for the benefit of the wider winter cereal research community.

## Reporting summary

Further information on research design is available in the Nature Portfolio Reporting Summary linked to this article.

### Supplementary information

Reporting Summary

### Source data

Source Data

## Data Availability

The plant materials analyzed in the present study are available from the corresponding authors upon request. The previously published sequences of genes discussed in this article can be found in NCBI GenBank and Ensembl Plants under the following accession numbers or gene identifiers: *Yr10*_*CG*_ (AF149112, TraesCS1B03G0000200), and *YrNAM* (OP490604, TraesCS1B03G0003600 LC.1 & TraesCS1B03G0003500 LC.1). Source data are provided with this paper.
