# Supplementary material for: The wheat stripe rust resistance gene YrNAM is Yr10
Source: Nat Commun. 2024 Apr 17;15:3291. doi: 10.1038/s41467-024-47513-z (PMC11024124; doi:10.1038/s41467-024-47513-z)
Supplement: Supplementary file 1 — Reporting Summary [file 41467_2024_47513_MOESM1_ESM.pdf]

nature portfolio

Corresponding author(s): Evans Lagudah, Peng Zhang, Robert McIntosh

Last updated by author(s): Mar 28, 2024

Reporting Summary

Nature Portfolio wishes to improve the reproducibility of the work that we publish. This form provides structure for consistency and transparency in reporting. For further information on Nature Portfolio policies, see our Editorial Policies and the Editorial Policy Checklist.

Statistics

For all statistical analyses, confirm that the following items are present in the figure legend, table legend, main text, or Methods section.

n/a	Confirmed	
☒	☐	The exact sample size (*n*) for each experimental group/condition, given as a discrete number and unit of measurement
☒	☐	A statement on whether measurements were taken from distinct samples or whether the same sample was measured repeatedly
☒	☐	The statistical test(s) used AND whether they are one- or two-sided<br>*Only common tests should be described solely by name; describe more complex techniques in the Methods section.*
☒	☐	A description of all covariates tested
☒	☐	A description of any assumptions or corrections, such as tests of normality and adjustment for multiple comparisons
☒	☐	A full description of the statistical parameters including central tendency (e.g. means) or other basic estimates (e.g. regression coefficient) AND variation (e.g. standard deviation) or associated estimates of uncertainty (e.g. confidence intervals)
☒	☐	For null hypothesis testing, the test statistic (e.g. *F*, *t*, *r*) with confidence intervals, effect sizes, degrees of freedom and *P* value noted<br>*Give P values as exact values whenever suitable.*
☒	☐	For Bayesian analysis, information on the choice of priors and Markov chain Monte Carlo settings
☒	☐	For hierarchical and complex designs, identification of the appropriate level for tests and full reporting of outcomes
☒	☐	Estimates of effect sizes (e.g. Cohen's *d*, Pearson's *r*), indicating how they were calculated

Our web collection on statistics for biologists contains articles on many of the points above.

Software and code

Policy information about availability of computer code

Data collection	No software used for data collection.

Data analysis	1. MutRenSeq - Contigs from the MutRenseq pipeline were used to identify (blast search) Yr10CG (AF149112) sequence using the following:<br>*CLC Genomics Workbench v10.0 (Qiagen, Hilden, Germany) (https://digitalinsights.qiagen.com/products-overview/ discovery-insights-portfolio/analysis-and-visualization/qiagen-clc-genomics-workbench/)<br>*CLC Sequence Viewer v8.0 (https://resources.qiagenbioinformatics.com/manuals/clcsequenceviewer/current/index.php?manual=Introduction_CLC_Sequence_Viewer.html)<br>*MutantHunter pipeline (https://github.com/steuernb)<br>*MuTrigo Python package (https://github.com/TC-Hewitt/MuTrigo)<br>*MYbaits protocol and the Triticeae NLR bait libraries (https://github.com/steuernb/MutantHunter/blob/master/Triticea_RenSeq_Baits_ V3.fasta.gz)<br>*BLASTn and SAMtools version 1.9.0<br><br>2. Yr10 mutant sequencing<br>Geneious Prime 2023.1.2 for quality trimming, assembly of wheat mutant Sanger sequencing reads and identification of base variants<br><br>3. Yr10 protein structure & conserved residues<br>*EVCoupling server (https://evcouplings.org/) to retrieve NAM domain-containing sequences and calculate amino acid conservation values<br>*AlphaFold 2.0 and PyMOL 2.5.7 to predict and view Yr10 structure

For manuscripts utilizing custom algorithms or software that are central to the research but not yet described in published literature, software must be made available to editors and reviewers. We strongly encourage code deposition in a community repository (e.g. GitHub). See the Nature Portfolio guidelines for submitting code & software for further information.

Data

Policy information about availability of data

All manuscripts must include a data availability statement. This statement should provide the following information, where applicable:

- Accession codes, unique identifiers, or web links for publicly available datasets
- A description of any restrictions on data availability
- For clinical datasets or third party data, please ensure that the statement adheres to our policy

The sequences of genes in this article can be found in NCBI GenBank and Ensembl Plants under the following accession numbers and gene identifiers: Yr10CG (AF149112, TraesCS1B03G000200) and YrNAM (OP490604, TraesCS1B03G0003600 LC.1 & TraesCS1B03G0003500 LC.1).

Research involving human participants, their data, or biological material

Policy information about studies with human participants or human data. See also policy information about sex, gender (identity/presentation), and sexual orientation and race, ethnicity and racism.

Reporting on sex and gender	None
Reporting on race, ethnicity, or other socially relevant groupings	None
Population characteristics	None
Recruitment	None
Ethics oversight	None

Note that full information on the approval of the study protocol must also be provided in the manuscript.

Field-specific reporting

Please select the one below that is the best fit for your research. If you are not sure, read the appropriate sections before making your selection.

☒ Life sciences ☐ Behavioural & social sciences ☐ Ecological, evolutionary & environmental sciences

For a reference copy of the document with all sections, see nature.com/documents/nr-reporting-summary-flat.pdf

Life sciences study design

All studies must disclose on these points even when the disclosure is negative.

Sample size	Sample size was determined according to standard procedures in wheat host-rust pathogen infection studies, typically 10-20 individuals per genotype against each of the two Pst isolates. The generated Avocet+Yr10 mutants were sequenced both by our lab, and also sent to Shandong Agricultural University for independent verification of the nucleotide changes in YrNAM by the laboratory of Ni et al.
Data exclusions	No data were excluded from analyses.

Replication	Mutant plants were identified in M2 generation and were confirmed by progeny test in M3 generation. All replication attempts successful.
Randomization	When phenotyping plants, we randomized mutant, wild-type, and control plants as is normal practice.
Blinding	Blinding was performed when phenotyping the mutant and wild-type lines and for their resistance to Pst.

Reporting for specific materials, systems and methods

We require information from authors about some types of materials, experimental systems and methods used in many studies. Here, indicate whether each material, system or method listed is relevant to your study. If you are not sure if a list item applies to your research, read the appropriate section before selecting a response.

Materials & experimental systems

n/a	Involved in the study
☒	☐ Antibodies
☒	☐ Eukaryotic cell lines
☒	☐ Palaeontology and archaeology
☒	☐ Animals and other organisms
☒	☐ Clinical data
☒	☐ Dual use research of concern
☐	☒ Plants

Methods

n/a	Involved in the study
☒	☐ ChIP-seq
☒	☐ Flow cytometry
☒	☐ MRI-based neuroimaging

Plants

Seed stocks	Lines Moro, YrVav7089 (backcross derivative of Triticum vavilovii AUS22498), T. spelta 415 (Plant Breeding Institute accession C89.19) Avocet S+Yr10 , Avocet S were all maintained at the Plant Breeding Institute, University of Sydney.
Novel plant genotypes	Six hundred seeds of wheat line Avocet S+Yr10 were mutagenized with 0.5% EMS. Four independent mutants were identified in M2 generation using the Yr10-avirulent race 239 E237 A-17+33+.
Authentication	The four mutants obtained (M6211, M6220, M6225 & M6227) were progeny tested and confirmed at the M3 generation.
